# Gender difference in the characteristics of and high-risk behaviours among non-injecting heterosexual methamphetamine users in Qingdao, Shandong Province, China

**DOI:** 10.1186/1471-2458-13-30

**Published:** 2013-01-14

**Authors:** Dianchang Liu, Zhenhong Wang, Tongsheng Chu, Shumin Chen

**Affiliations:** 1 Shandong Clinical College of Skin Diseases, Anhui Medical University, 27397, Jingshi Lu, Jinan, Shandong, 250022, China; 2 The Third People's Hospital of Chengyang, Wangsha Lu, Chengyang, Qingdao, 266107, China; 3 Shandong Provincial Institutes of Dermatology and Venerology, 27397, Jingshi Lu, Jinan, 250022, China

**Keywords:** Methamphetamines, Heterosexual, High-risk behaviour, Gender

## Abstract

**Background:**

Despite the increasing risk of HIV infections, few studies concerning the characteristics of non-injecting heterosexual methamphetamine (MA) users and related risk behaviours have been conducted in China.

**Methods:**

Gender differences in socio-demographic characteristics, perception of MA and STD/HIV, MA use practices, and sexual behaviours related to MA use were examined among 398 non-injecting heterosexual MA users (288 males, 110 females).

**Results:**

Male MA users were more likely to be married, local, and self-employed; female MA users were more likely to be young, single, engaged in commercial service or unemployed. Female MA users usually start MA use at an earlier age than males (24.3 vs. 31.3 years old), with shorter abuse durations (2.6 vs. 2.9 years), higher frequency of MA use (3.6 vs. 2.4 times per week), and higher likelihood of using MA with heterosexual partners (100% vs. 78.1%). More male MA users have had multiple sex partners (96.9% vs. 77.3%) and sex exchanges (72.9% vs. 46.4%). Among 277 males who had had sex with commercial sex workers (CSW), 69.4% never used condoms, and among 77 males who had had sex with multiple partners who are commercial sex workers and always or usually used condoms, 87.0% never changed condoms when changing partners.

**Conclusion:**

There may be gender difference in the characteristics of high-risk behaviours among non-injecting heterosexual MA users. The findings suggest the integration of specific risk reduction strategies into intervention programs for non-injecting heterosexual MA user populations may significantly improve program goals.

## Background

The use of amphetamine-type stimulants (ATS) including methamphetamines (MA) is a growing global health problem. ATS are the second most widely used group of substances, among which MA is the most prominent. It is estimated globally that the annual prevalence for ATS ranged between 0.3% and 1.2% in 2010, or some 14 to 52 million people aged 15 years to 64 years who had used such substances at least once in 2010 [1].

Many studies have addressed the physical and psychological harms of chronic MA use [2,3] and its association with high-risk sexual behaviours among men who have sex with men (MSM) [4,5]. MA use among MSM has been associated with low rates of condom use, high rates of unprotected anal sex, prolonged sexual activity, multiple partners, and casual partners [6-8]. Some studies among female drug users have also shown MA use to be associated with elevated concomitant sexual risks, including a higher number of sexual partners, unprotected vaginal and/or anal sex, and exchanging sex for money or drugs [9-11]. Besides increasing high-risk sexual behaviours, MA use can enhance HIV-1 infections in human macrophages in vitro [12]. Many studies indicate high risk of HIV and other sexually transmitted disease (STDs) infections among different MA users [13-16].

China is one of two countries with the highest records of ATS abuse. By the end of 2011, 1.794 million drug users had been registered in the updated Drug Abusers Information Database of China. Users of “new-type drugs,” including ATS and ketamine, accounted for 32.7% of the total number of registered drug users [17].

Injection drug use (IDU), which mainly includes heroin, used to be the major cause of HIV infections, and the association between IDU and risk of HIV infections has been well documented [18]. However, the transmission routes of HIV have largely changed in recent years. The proportion of cases resulting from sexual transmission increased from 33.1% in 2006 to 76.3% in 2011 [19]. Compared with non-MA users, non-injecting heterosexual MA users report significantly higher numbers of sex partners, more frequent engagements in anal sex, and less frequent use of condoms and are twice as likely to have sex with a commercial sex worker (CSW) or exchange sex for drugs [7].

To date, despite the increasing risk for widespread HIV infections, little attention has been given to non-injecting heterosexual MA users, and the characteristics of non-injecting heterosexual MA users and related risk behaviours among them have not been well studied in China. A pilot study in Shandong Province found an alarmingly high prevalence of MA use among female CSWs. MA users were more likely to be inconsistent in condom usage and have syphilis [20]. The aim of this study is to explore the characteristics of male and female non-injecting heterosexual MA users in China and evaluate their high-risk behaviours in order to find specific intervention measures to prevent STDs and HIV infection.

## Methods

### Study site

The study was conducted in Chengyang District, Qingdao, Shandong Province, China. Qingdao is a coastal city in Shandong that attracts many foreign and domestic investors and tourists because of its mild climate, convenient transportation, and favourable policies. However, accompanied by booms in economic development and the entertainment industry, drug trafficking and abuse have become increasingly common in the city over the last ten years. Famous for “Iceland” in China (Qingdao is on the eastern end of Shandong Peninsula, and MA looks like ice in appearance), aside from having many MA users, Qingdao is one of the distribution centres of MA from South Korea, Japan, and Russia to China. Chengyang is one of the suburban districts of Qingdao. It connects the urban and rural areas of northern Qingdao and has a total area of 553.2 square kilometres and a population of 0.76 million people, including a mobile population of 0.3 million. It is one of the major industrial areas in Qingdao and, for many years, has been among the top districts in Shandong Province for exports. In the district, there are many foreign-owned or joint enterprises, manufacturing, and entertainment venues. MA users can be found in nightclubs, KTVs (one kind of entertainment venues with many private rooms where customers can sing and dance by themselves), bars, bath centres, hotels, and so on.

### Recruitment and screening of participants

This study was conducted as part of the “AIDS Projects in the Cover” program funded by the Bill & Melinda Gates Foundation and the Chinese government. Most participants were recruited from nightclubs, KTVs, bars and bath centres, accounting for 22.3%, 30.5%, 30.3%, and 9.6%, respectively, of the sample. Community outreach workers visited these venues and made the acquaintance of potential study subjects. The following inclusion criteria were used: (1) self-identified heterosexual; (2) use of MA as a major addictive drug; (3) use of MA by any means aside from injection; (4) in the 1 month prior to screening, had used MA at least once. A person satisfying all of these criteria was asked for an interview and examination at an STD clinic in the community hospital the following day. The “Snow Ball” approach was also applied in enrolling participants. Around 5.1% of the participants were recruited directly by doctors at the STD clinic, and 2.3% were referred by bosses of entertainment venues, friends, and STD clinical attendees. All surveys and examinations were conducted at the STD clinic in the community hospital by an STD doctor and a nurse. Around 87.5% of the female participants were CSWs; the remaining female participants were girlfriends or casual sex partners of male participants.

### Data collection

The research protocol was approved by the Ethical Committee of Shandong Provincial Hospital for Skin Diseases, and all participants completed verbal informed consent prior to participation. An interviewer-administered questionnaire was used in data collection (see Additional file 1 for the questionnaire). The following characteristics of MA users were examined: demographics (gender, age, marital status, ethnicity, education, residency and employment); perception of MA; perception of STDs/HIV; MA use; and sexual behaviour related to MA use. Each topic area was covered by several variables, each in the form of a question. Those diagnosed with STDs were given free and appropriate treatments.

### Statistical analysis

The characteristics of MA users by gender were examined. Differences in demographic characteristics, perception of MA, perception of STDs/HIV, MA use, and sexual behaviour related to MA use between males and females were compared. Chi-square and t-tests were used for statistical analysis.

## Results

### Demographic information

As shown in Table 1, among the 398 participants, 288 were males and 110 were females. The average age of females was significantly lower than males (27.0 vs. 34.2 years old). Males were more likely to be married (70.5% vs. 40.9%), while females were more likely to be single (50.0% vs. 25.3%). Males were more likely to be permanent residents (86.5% vs. 48.2%), while females were more likely to be in the mobile population (51.8% vs. 13.5%). Males were more likely to be self-employed businessmen (73.3% vs. 11.8%), while females were more likely to be engaged in commercial service (56.6% vs. 4.9%) or unemployed (30.9% vs. 16.3%). All of the differences between male and female MA users were statistically significant (p<0.05). No significant differences were observed in terms of ethnicity and education level by gender, with Han accounting for 88.7% and middle school education accounting for 69.3% of the sample, respectively.

**Table 1:** Demographic differences among 398 participants by gender

**Variable**	**Total (N=398)**	**Male (N=288)**	**Female (N=110)**	**t**	**X**^**2**^	**P value**
Age, M (SD), range	32.3 (9.6), 16-67	34.2 (1.0), 16-67	27.0 (6.3), 19-48	7.11		<0.05
Marital status						
Single, n (%)	128 (32.2)	73 (25.3)	55 (50.0)		38.302	<0.05
Married, n (%)	256 (64.3)	203 (70.5)	53 (40.9)			
Divorced, n (%)	14 (3.5)	12 (4.2)	2 (9.1)			
Ethnicity						
Han, n (%)	353 (88.7)	262 (91.0)	91 (82.7)		5.772	>0.05
Korean, n (%)	26 (6.5)	14 (4.9)	12 (10.9)			
Others, n (%)	19(4.8)	12 (4.2)	7 (6.4)			
Education						
Elementary school, n (%)	18 (4.5)	15 (5.2)	3 (2.7)		1.671	>0.05
Middle school, n (%)	276 (69.3)	201 (69.8)	75 (68.2)			
High/technical school, n (%)	92 (23.1)	64 (22.2)	28 (25.5)			
College or above, n (%)	12 (3.0)	8 (2.8)	4 (3.6)			
Residency						
Permanent residents, n (%)	302 (75.9)	249 (86.5)	53 (48.2)		63.717	<0.05
Mobile population, n (%)	96 (24.1)	39 (13.5)	57 (51.8)			
Employment						
Self-employed, n (%)	224 (56.3)	211 (73.3)	13 (11.8)		177.11	<0.05
Commercial service, n (%)	76 (19.1)	14 (4.9)	62 (56.4)			
Unemployed, n (%)	81 (20.4)	47 (16.3)	34 (30.9)			
Others, n (%)	17 (4.3)	16 (5.6)	1 (0.9)			

### Perception of MA use

As shown in Table 2, 92.2% of the respondents considered MA use as common as smoking and this perception was more acceptable in males than in females (96.2% vs. 81.8%, p<0.05). Most males and females believed that MA enhances sexuality (97.2% vs. 99.1%, p>0.05) while more males than females believed that it helps promote anti-inebriation (63.9% vs. 37.3%, p<0.05) and pain relief (44.1% vs. 33.6%, p<0.05). No gender differences were found in terms of perception of addictiveness of MA (47.2% vs. 44.5%, p>0.05) and proportion of self-reported addiction to MA (46.2% vs. 43.6%, p>0.05). Among those who admitted MA addiction, 30.1% of the males responded that it had occurred to them to abstain from MA and wanted to try it. In comparison, 33.3% of the females responded that it had occurred to them to abstain from MA but only 8.3% of them wanted to try it (p<0.05).

**Table 2:** Perceptions of MA use among MA users

**Questions**	**Total (%, N=398)**	**Male (%, N=288)**	**Female (%, N=110)**	**X**^**2**^	**P value**
Do you think MA use is as common as smoking? (Yes)	367 (92.2)	277 (96.2)	90 (81.8)	22.861	<0.05
Can MA enhance sexuality? (Yes)	389 (97.7)	280 (97.2)	109 (99.1)		>0.05
Does MA have the function of anti-inebriation? (Yes)	225 (56.5)	184 (63.9)	41 (37.3)	34.120	<0.05
Can MA relieve pain? (Yes)	164 (41.2)	127 (44.1)	37 (33.6)	13.287	<0.05
Is MA addictive? (Yes)	185 (46.5)	136 (47.2)	49 (44.5)	0.229	>0.05
Are you addicted to MA? (Yes)	181 (45.5)	133 (46.2)	48 (43.6)	0.208	>0.05
Did it occur to you to abstain from MA? (Yes)	56/181(30.9)	40/133 (30.1)	16/48 (33.3)	0.157	>0.05
Do you want to have a try to abstain from MA? (Yes)	44/181(24.3)	40/133 (30.1)	4/48 (8.3)	9.061	<0.05

### Perception of STDs/AIDS

As shown in Table 3, 99.7% of the males and 100% of the females had heard of syphilis (p>0.05). The proportion of those who claimed knowing the transmission routes of syphilis was higher in males than in females (93.1% vs. 77.3%, p<0.05). Although as high as 99.3% of the males and 100% of the females reported knowing the transmission routes of HIV, 64.2% of the males and 84.5% of the females believed that HIV can be transmitted by eating together (p>0.05). Approximately 97.2% of the males and 100% of the females knew the risk of multiple sex partners. However, 33.3% of the males and 29.1% of the females believed that an HIV-infected person could be identified by appearance, and only 52.4% of the males and 67.3% of the females believed that STDs/AIDS could be prevented by using condoms (p<0.05).

**Table 3:** Perceptions of STD/AIDS among MA users

**Questions**	**Total (%, n=398)**	**Male (%, n=288)**	**Female (%, n=110)**	**X**^**2**^	**P value**
Have you ever heard of syphilis? (Yes)	397 (99.7)	287 (99.7)	110 (100)		
Do you know the transmission routes of syphilis? (Yes)	353 (88.7)	268 (93.1)	85 (77.3)	19.772	<0.05
Do you know the transmission routes of HIV? (Yes)	396 (99.5)	286 (99.3)	110 (100)		
Can HIV be transmitted by eating together? (Yes)	278 (69.8)	185 (64.2)	93 (84.5)	17.978	<0.05
Do you know the risk of multiple sex partners? (Yes)	390 (98.0)	280 (97.2)	110 (100)		>0.05
Can HIV infection be identified by appearance? (Yes)	128 (32.2)	96 (33.3)	32 (29.1)	1.262	>0.05
Can STD/AIDS be prevented by using condom? (Yes)	225 (56.5)	151 (52.4)	74 (67.3)	17.800	<0.05

### Behaviours of MA use

As shown in Table 4, most of the male and female respondents reported MA as the only drug they used (94.8% vs. 98.2%, p>0.05). Both the average onset age and the average years of MA use among females were lower than those among males (24.3 vs. 31.3, p<0.05; 2.6 vs. 2.9, p<0.05), and more females started MA use at an early age (X^2^=62.170, p<0.05) with a shorter period of use (X^2^=19.411, p<0.05) than males. However the average times of MA use per week was higher among females than among males (3.6 vs. 2.4, p<0.05), and the proportion of those who used MA an average of 5 times and above per week was much higher in females than in males (X^2^=89.468, p<0.05). MA was usually used in groups, and the group size reported by males was larger than that reported by females (4.2 vs. 3.8 persons, p<0.05). Females were more likely to use MA with heterosexual partners than males (100% vs. 78.1%, p<0.05).

**Table 4:** MA use behaviours among MA users

**Variable**	**Total (N=398)**	**Male (N=288)**	**Female (N=110)**	**t**	**X**^**2**^	**P value**
Using MA only, n (%)	381 (95.7)	273 (94.8)	108 (98.2)			>0.05
Onset age of MA use, M (SD), range	29.4 (9.1), 16-62	31.3 (9.3), 16-67	24.3 (6.2), 17-44	7.367		<0.05
<20 years old	42 (10.6)	13 (4.5)	29 (26.4)			
20~29 years old	174 (43.5)	116 (40.3)	58 (52.7)		62.170	<0.05
30~39 years old	120 (29.9)	100 (34.7)	20 (18.2)			
≥40 years old	62 (16.1)	59 (20.5)	3 (2.7)			
Years of MA use, M (SD), range	2.8 (1.1), 1-8	2.9 (1.2), 1-8	2.6 (0.9), 1-5	2.650		<0.05
1 year	42 (10.6)	34 (11.8)	8 (7.3)			
2 years	127 (31.9)	75 (26.0)	52 (47.3)			
3 years	128 (32.2)	96 (33.3)	32 (29.1)		19.411	<0.05
4 years	71 (17.8)	56 (19.4)	15 (13.6)			
≥5 years	30 (7.5)	27 (9.4)	3 (2.7)			
Times of MA use per week, M (SD), range	2.8 (1.4), 1-7	2.4 (1.0), 1-6	3.6 (1.9), 1-7	8.270		<0.05
1 time	44 (11.1)	33 (11.5)	11 (10.0)			
2 times	183 (46.0)	150 (52.1)	33 (30.0)			
3 times	74 (18.6)	62 (21.5)	12 (10.9)		89.468	<0.05
4 times	53 (13.3)	37 (12.8)	16 (14.5)			
≥5 times	44 (11.1)	6 (2.1)	38 (34.5)			
Number of persons using MA together (SD), range	4.1 (1.6), 2-10	4.2 (1.4), 2-10	3.8 (1.9), 2-10	2.409		<0.05
Always using MA with heterosexual partners, n (%)	335 (84.2)	225 (78.1)	110 (100)		28.588	<0.05

### High-risk sexual behaviours related to MA use

As shown in Table 5, the proportions in males who had had sex with multiple partners and exchanged sex partners during MA use was significantly higher than those in females (96.9% vs. 77.3%, X^2^=39.147, p<0.05; 72.9% vs. 46.4%, X^2^=24.862, p<0.05). Among 288 male respondents, 96.2% had had sex with CSWs during MA use, and the number of CSWs as sex partners per MA use was 1.9 persons. Among 277 males who had had sex with CSWs, 72.2% had never used condoms. Among 77 males who had had sex with multiple CSW partners and reported always or usually using condoms, 87.0% had never changed condoms when changing CSW partners. About 96.4% of the females reported having had sex with partners for MA or money.

**Table 5:** High-risk sexual behaviours related to MA use among MA users

**Variable**	**Total (n=398)**	**Male (n=288)**	**Female (n=110)**	**t**	**X**^**2**^	**P value**
Had sex with multiple partners during MA use	364 (91.5)	279 (96.9)	85 (77.3)		39.147	p<0.001
Exchanged sex partners during MA use	261 (65.6)	210 (72.9)	51 (46.4)		24.862	p<0.001
Had sex with commercial sex workers(CSWs) during MA use		277 (96.2)				
Number of CSWs as sex partners per MA use, M (SD), range		1.9 (0.7), 1-4				
Condom use in sex with CSWs						
Never		200 (72.2)				
Usually		58 (20.9)				
Always		19 (8.9)				
Changing condoms when changing CSWs						
Never		67 (87.0)				
Usually		3 (3.9)				
Always		7 (9.1)				

## Discussion

Gender differences among MA users have been reported by some studies. Female MA users were more likely to be younger, have lower educational level, have never been married, and be an MA-using sex partner [21-23]. Our findings show significant differences between the demographic characteristics of male and female MA users, and some new findings in this study differed from those in other studies. Most male MA users in this study were married and local or self-employed businessmen; most female MA users were young, single, and mobile CSWs. These findings are interesting because they suggest that gender-specific intervention measures should be implemented in risk reduction programs for MA user populations.

The reasons for MA use were investigated in this survey. The major reason for MA use is its benefit in enhancing sexuality; most male and female respondents neglected its addictiveness. Furthermore, both males and females had several wrong perceptions of MA. MA was usually regarded as a daily life necessity, very much like cigarettes, especially by male respondents. This finding indicates that more information on the risks and harm of MA should be provided in intervention programs in the future. MA use is a complicated social problem in many countries. The reasons why more and more people fall into MA addiction require more studies from the physiological, psychological, and social perspectives. Individual, social, and environmental factors have been reported to be associated with initiating MA injection [24]. In this study, among those who recognised its addictiveness, fewer females than males wanted to try to abstain from MA. The reasons underlying this behaviour require further investigation.

Gender differences were observed in terms of MA use behaviours. Most female MA users initiated MA use at an early age, had shorter abuse durations, used MA more frequently, and were more likely use MA with heterosexual partners. MA enhances sexual performance, sensitivity, and pleasure, increasing the risk of trauma from prolonged intercourse and failure to use condoms [25,26], and is associated with having multiple sex partners and unprotected sex [9-11]. Results from this paper support previous findings showing that MA use can greatly increase the risk of STD/HIV transmission. Although most respondents in this study had enough knowledge of STD/AIDS, some misunderstandings were observed. Most male and female respondents recognised the risk of multiple sex partners; however, many MA users did not know how to prevent STDs/AIDS using condoms. High-risk sexual behaviours related to MA use among these respondents were very common, including having sex with multiple partners and exchanging sex partners. More males had been engaged in group sex and sex partner exchanges than females because most male respondents had had sex with CSWs during MA use. It indicates the truth that many sex episodes involved one man and more than one woman including those women who are not MA users during MA use, suggesting that some form of intervention specific to these kinds of sex encounters should be conducted. Furthermore, many males had also been engaged in other high-risk sexual practices, such as never using condoms and never changing condoms when changing CSW partners, which may endanger their female partners as well.

Several limitations are present in this study, including the sample population and the data collection methods. First, the study utilised non-random sampling recruitment methods and had inclusion criteria confined to non-injected heterosexual MA users; thus, the generalisability of the study results could be limited. Second, behavioural data were collected through self-reporting and memory or recall of behaviours during sexual or MA use events may be problematic. Third, most female MA users recruited into the study report behaviors that would lead them to be classified as sex workers. Therefore, the sample tells us little about MA use and related knowledge, attitudes, and risk behaviors in the broader population of women who use MA without injection. Finally, although STD/HIV testing was conducted for each participant in this cross-sectional study, detailed information about actual STD/HIV infection rates in this article was not provided. A multi-factor analysis on STD/HIV prevalence among non-injecting heterosexual MA users will be conducted in another study.

The study was characterised by a number of strengths. First, this study provides the first report in China addressing the characteristics of and high-risk behaviours among non-injecting heterosexual MA users. Second, the findings enrich our knowledge of gender differences in demographic characteristics, perception of MA and STDs/HIV, and high-risk behaviours among non-injecting heterosexual MA users. Third, this study discussed the reasons for MA use, although further investigation is needed.

## Conclusion

In conclusion, gender differences in the characteristics of and high-risk behaviours among non-injecting heterosexual MA users were observed. The findings suggest that integrating specific risk reduction strategies for non-injecting heterosexual MA user populations into intervention programs will be beneficial to the success of such programs.

## Abbreviations

ATS: Amphetamine-type stimulants; MA: Methamphetamines; MSM: Men who have sex with men; STDs: Sexually transmitted diseases; IDU: Injection drug use; CSWs: Commercial sex workers.

## Competing interests

No competing interests declared.

## Authors’ contributions

SC participated in conception, revision and finalization of the manuscript. DL carried out the studies, participated in design, data collection and writing of the draft. ZW carried out participants recruitment, data collection and data analysis. TC participated in data collection and revision. All authors read and approved the final manuscript.

## Pre-publication history

The pre-publication history for this paper can be accessed here:

http://www.biomedcentral.com/1471-2458/13/30/prepub

## Supplementary Material

Additional file 1 Questionnaire given to MA users. Click here for file

## References

[B1] United Nations Office on Drugs and Crime World Drug Report 2012 New York: United Nations [http://www.unodc.org/documents/data-and-analysis/WDR2012/WDR_2012_web_small.pdf]

[B2] Buxton JA Dove NA The burden and management of crystal meth use Can Med Assoc J 2008 178 1537 1539 10.1503/cmaj.071234 18519899 PMC2396355

[B3] Darke S Kaye S McKetin R Duflou J Major physical and psychological harms of methamphetamine use Drug Alcohol Rev 2008 27 253 262 10.1080/09595230801923702 18368606

[B4] Halkitis PN Parsons JT Stirratt MJ A double epidemic: Crystal methamphetamine drug use in relation to HIV transmission among gay men J Homosex 2001 41 17 35 10.1300/J082v41n02_02 11482426

[B5] Prestage G Degenhardt L Jin F Grulich A Imrie J Kaldor J Kippax S Predictors of frequent use of amphetamine type stimulants among HIV-negative gay men in Sydney, Australia Drug Alcohol Depend 2007 91 260 268 10.1016/j.drugalcdep.2007.06.009 17640831 PMC2699371

[B6] Gorman M A tale of two epidemics: HIV and stimulant use Focus San Francisco Calif 1998 13 1 3 11365170

[B7] Molitor F Truax SR Ruiz JD Sun RK Association of methamphetamine use during sex with risky sexual behaviors and HIV infection among non-injection drug users West J Med 1998 168 93 97 9499742 PMC1304836

[B8] Paul JP Stall R Davis F Sexual risk for HIV transmission among gay/bisexual men in substance-abuse treatment AIDS Educ Prev 1993 5 11 24 8481269

[B9] Lorvick J Martinez A Gee L Kral AH Sexual and injection risk among women who inject methamphetamine in San Francisco J Urban Health 2006 83 497 505 10.1007/s11524-006-9039-4 16739050 PMC2527191

[B10] Semple SJ Grant I Patterson TL Female methamphetamine users: social characteristics and sexual risk behavior Women Health 2004 40 35 50 10.1300/J013v40n02_03 15829444

[B11] Shannon K Strathdee S Shoveller J Zhang R Montaner J Tyndall M Crystal methamphetamine use among female street-based sex workers: Moving beyond individual-focused interventions Drug Alcohol Depend 2011 113 76 81 10.1016/j.drugalcdep.2010.07.011 20810223 PMC3392206

[B12] Liang H Wang X Chen H Song L Ye L Wang SH Wang YJ Zhou L Ho WZ Methamphetamine enhances human immunodeficiency virus infection of macrophages Am J Pathol 2008 172 1617 1624 10.2353/ajpath.2008.070971 18458095 PMC2408421

[B13] Sherman SG Sutcliffe CG Srirojn B German D Thomson N Aramrattana A Celentano DD Incidence of HIV and sexually transmitted infections and risk factors for acquisition among young methamphetamine users in northern Thailand Sex Transm Dis 2009 36 284 289 10.1097/OLQ.0b013e318191ba17 19295472 PMC3682416

[B14] Cartier JJ Greenwell L Prendergast ML The persistence of HIV risk behaviors among methamphetamine-using offenders J Psychoactive Drugs 2008 40 437 446 10.1080/02791072.2008.10400650 19283948 PMC3286359

[B15] Buchacz K McFarland W Kellogg TA Loeb L Holmberg SD Dilley J Klausner JD Amphetamine use is associated with increased HIV incidence among men who have sex with men in San Francisco AIDS 2005 19 1423 1424 10.1097/01.aids.0000180794.27896.fb 16103774

[B16] Plankey MW Ostrow DG Stall R Cox C Li X Peck JA Jacobson LP The relationship between methamphetamine and popper use and risk of HIV seroconversion in the multicenter AIDS cohort study J Acquir Immune Defic Syndr 2007 45 85 92 10.1097/QAI.0b013e3180417c99 17325605 PMC3486782

[B17] National Narcotic Control Commission China police achieve success in anti-drug actions [http://news.xinhuanet.com/english/china/2012-06/25/c_123328672.htm]

[B18] The State Council AIDS Working Committee Office and the UN Theme Group on HIV/AIDS in China A joint assessment of HIV/AIDS prevention, treatment and care in China [http://www.undp.org.cn/downloads/otherlocal/HIV/20080104.pdf]

[B19] Ministry of Health of the People’s Republic of China 2012 China AIDS Response Progress Report [http://www.aidsdatahub.org/dmdocuments/UNGASS_2012_China_Narrative_Report.pdf]

[B20] Liao M Jiang Z Zhang X Kang D Bi Z Liu X Fu J Zhang N Mao W Jiang B Jia Y Syphilis and Methamphetamine Use Among Female Sex Workers in Shandong Province China. Sex Transm Dis 2011 38 57 62 10.1097/OLQ.0b013e3181ebb475 20679964

[B21] Cheng WS Garfein RS Semple SJ Strathdee SA Zians JK Patterson TL Differences in Sexual Risk Behaviors among Male and Female HIV-Seronegative Heterosexual Methamphetamine Users Am J Drug Alcohol Abuse 2009 35 295 300 10.1080/00952990902968585 19591066 PMC2913870

[B22] Senjo SR Trafficking in meth: an analysis of the differences between male and female dealers J Drug Educ 2005 35 59 77 10.2190/966Q-R6Y3-7G08-DTP8 16270698

[B23] Lin SK Ball D Hsiao CC Chiang YL Ree SC Chen CK Psychiatric comorbidity and gender differences of persons incarcerated for methamphetamine abuse in Taiwan Psychiatry Clin Neurosci 2004 58 206 212 10.1111/j.1440-1819.2003.01218.x 15009828

[B24] Marshall BD Wood E Shoveller JA Buxton J Montaner J Kerr T Individual, Social, and Environmental Factors Associated with Initiating Methamphetamine Injection: Implications for Drug Use and HIV Prevention Strategies Prev Sci 2011 12 173 180 10.1007/s11121-010-0197-y 21274628 PMC3107866

[B25] Potula R Persidsky Y Adding fuel to the fire: Methamphetamine enhances HIV infection Am J Pathol 2008 172 1467 1470 10.2353/ajpath.2008.080130 18458093 PMC2408407

[B26] National Institute on Drug Abuse Methamphetamine Abuse and Addiction [http://www.drugabuse.gov/PDF/RRMetham.pdf]

