# Supplementary material for: Gender difference in the characteristics of and high-risk behaviours among non-injecting heterosexual methamphetamine users in Qingdao, Shandong Province, China
Source: BMC Public Health. 2013 Jan 14;13:30. doi: 10.1186/1471-2458-13-30 (PMC3585885; doi:10.1186/1471-2458-13-30)
Supplement: Additional file 1 — Questionnaire given to MA users. [file 1471-2458-13-30-S1.pdf]

Attachment 1. Questionnaire given to MA users

Questionnaire of MA users

Informed consent: *I am Dr. X, working for the XX. I am doing a study on health problem of methamphetamine users. I am going to give you information and invite you to be part of this research. STD and AIDS have been spreading among methamphetamine users. We want to find ways to stop this from happening. We believe that you can help us by telling us what you know about methamphetamine, and about your concepts and behaviours related to STD and AIDS. This study will involve your participation in an interview that will take about half an hour. Your participation in this research is entirely voluntary. It is your choice whether to participate or not. We also want to know how serious STD and AIDS are spreading in this population, so after the interview you are welcome to take an examination in our clinic. All the services provided are free and you can come anytime. If you choose not to participate all the services you receive at our clinic will continue and nothing will change. We are asking you to share with us some very personal and confidential information, and you may feel uncomfortable talking about some of the topics. During the interview, if you do not wish to answer any of the questions during the interview, you may say so and I will move on to the next question. There will be no direct benefit to you, but your participation is likely to help us find out more about how to prevent and control STD and AIDS in the population. The information recorded is confidential, and no one else except our work staff will access to the information documented during your interview. If you have any questions, you can ask us now or later. If you wish to ask questions or have an examination later, you may contact Dr. Wang Zhenhong, the Third People's Hospital of Chengyang, his telephone number is13853289000.*

A--registration

Registration Number □□□

A01 Date of interview year□□□□month□□date□□

A02 Sites of recruitment

①Nightclubs ②KTVs ③bars ④bath centres ⑤STD clinic ⑥hotels ⑦tea houses □

B--General information

B01 Sex ①Male ②Female □

B02 Birthday year □□□□month□□

B03 Marital status ①Single ②Married ③Divorced □

B04 Ethnicity ①Han ②Korean ③Others □

B05 Education

①Elementary school ②Middle school ③High/technical school ④College or above □

B06 Residency ①Permanent residents ②Mobile population □

B07 Employment

① Self-employed ②Commercial service ③Unemployed ④Others □

C-- **Perception of MA use**

C01 Do you think MA use is as common as smoking? ①Yes ②No ③Dot not know □

C02 Can MA enhance sexuality? ①Yes ②No ③Dot not know □

C03 Does MA have the function of anti-inebriation? ①Yes ②No ③Dot not know □

C04 Can MA relieve pain? ①Yes ②No ③Dot not know □

C05 Is MA addictive? ①Yes ②No ③Dot not know □

C06 Are you addicted to MA? ①Yes ②No ③Dot not know □

C07 Did it occur to you to abstain from MA? ①Yes ②No □

C08 Do you want to have a try to abstain from MA? ①Yes ②No □

D- Perception of STD/AIDS

D01 Have you ever heard of syphilis? ①Yes ②No □

D02 Do you know the transmission routes of syphilis? ①Yes ②No □

D03 Do you know the transmission routes of HIV? ①Yes ②No □

D04 Can HIV be transmitted by eating together? ①Yes ②No ③Dot not know □

D05 Do you know the risk of multiple sex partners? ①Yes ②No □

D06 Can HIV infection be identified by appearance? ①Yes ②No ③Dot not know □

D07 Can STD/AIDS be prevented by using condom? ①Yes ②No ③Dot not know □

E--Behaviours of MA use

E01 Did you use other drugs except for MA? ①Yes ②No □

E02 How old was you when you first used MA? □□
E03 How many years have you used MA? □□
E04 How many times have you used per week in average in the past year? □□
E05 How many persons in average have you used together in the past year? □□
E06 How often did you use MA with heterosexual partners?

① Every time ②Sometimes ③Never □

F—High-risk sexual behaviours related to MA use

F01 Have you ever had sex with multiple partners during MA use? ①Yes ②No □

If yes

F02 Have you ever exchanged sex partners each other during MA use? ①Yes ②No □

F03 Have you ever had sex with partners for MA or money?(female) ①Yes ②No □

F04 Have you had sex with CSWs during MA use?(male) ①Yes ②No □

If yes
F05 How many CSWs did you usually have sex per MA use? □□
F06 How often have you used condom when having sex with CSWs?
① Never ②Usually ③Always □
F07 How often have you changed condoms when changing CSWs
① Never ②Usually ③Always □
